# Beyond the test: patient-reported concerns and worries regarding kidney conditions and *APOL1* genetic testing among Black patients

**DOI:** 10.3389/fneph.2026.1815147

**Published:** 2026-07-22

**Authors:** Rengin Elsurer Afsar, Bryan Clair, Aliza Anwar Memon, John C. Edwards, Kana N. Miyata, Baris Afsar, Mark Schnitzler, Huiling Xiao, Amber Carriker, Fadee Abu Al Rub, Chi-yuan Hsu, Anthony N. Muiru, Barry I. Freedman, Marie D. Philipneri, Yasar Caliskan, Krista L. Lentine, St. Louis APOL1 Study Collaborators

**Affiliations:** 1 SSM Health Saint Louis University Hospital, St. Louis, MO, United States; 2 Saint Louis University School of Medicine, St. Louis, MO, United States; 3 Department of Mathematics, Saint Louis University, St. Louis, MO, United States; 4 Mid-America Transplant, St. Louis, MO, United States; 5 University of California, San Francisco, San Francisco, CA, United States; 6 Wake Forest University School of Medicine, Winston-Salem, NC, United States

**Keywords:** African Americans, apolipoprotein L1, chronic kidney disease, genetic testing, patient concerns, risk factors

## Abstract

**Background:**

Apolipoprotein L1 (*APOL1*) renal risk variants (RRVs) contribute to the increased risk of kidney disease among Black Americans, but use of genetic testing may be limited by patient concerns regarding clinical implications and effects on family.

**Methods:**

Black adults were prospectively enrolled for *APOL1* genotyping, surveys, and clinical record review beginning in January 2019 at a Midwestern U.S. medical center (NCT05656261). Patient-reported concerns about kidney conditions and potential worry related to having an *APOL1* high-risk genotype were analyzed at baseline and 3-months.

**Results:**

Among 200 participants enrolled with completed 3-month follow-up as of January 2025, 17.5% (35/200) carried an *APOL1* high-risk genotype (2 RRVs). At baseline, concern about kidney conditions was common: 39% reported being extremely concerned, 34% very concerned, 12% somewhat concerned, 10% a little concerned, and 6% not concerned at all. Higher baseline concern was observed among women and those with a family history of both kidney disease and hypertension. At 3 months, concern levels were unchanged in 45% of participants, decreased in 36%, and increased in 19%. *APOL1* high-risk genotypes were more common in those with an increase, compared to a decrease, in kidney concern at follow-up (29% vs 11%; P = 0.02). Worry related to having an *APOL1* high-risk genotype declined over time, from 14% at baseline to 8% at 3 months.

**Conclusion:**

In this prospective cohort, patient-reported concerns about kidney conditions were common and generally stable, with higher levels observed in certain subgroups. Although *APOL1* high-risk genotypes were more common among participants whose kidney concerns increased than among those whose concerns decreased, overall patient-reported worry about genetic risk declined. These findings may help inform patient education and counseling surrounding *APOL1* testing.

## Introduction

The frequency of chronic kidney disease (CKD) in Black individuals is two to three times higher than in White Americans. A portion of this increased risk appears attributable to polymorphisms in the gene encoding apolipoprotein L1 (*APOL1*) (1, 2). The two renal risk variants (RRVs), G1 (two amino acid substitutions) and G2 (two amino acid deletions), evolved to confer resistance to certain subspecies of *Trypanosoma* parasites. Combinations of two RRVs (G1/G1, G2/G2, or G1/G2) are referred to as *APOL1* high-risk genotypes and are associated with increased risk of CKD development and progression (1). Many individuals with *APOL1*-associated kidney disease and subnephrotic proteinuria are misclassified as having CKD of unknown etiology or hypertension-attributed CKD (2). In the United States, the estimated allelic frequency among Black Americans is 20%-22% for G1 and 13%-15% for G2, with approximately 10%-15% carrying an *APOL1* high-risk genotype (1). Despite its clinical relevance for characterizing CKD etiology and risk, *APOL1* genetic testing remains uncommon among Black patients with established CKD or at risk for kidney complications.

Patients with or at risk for CKD may experience concern related to uncertainty about their health and potential long-term complications, including the need for kidney replacement therapy (3, 4). In patients with or at risk for CKD, genetic diagnosis may offer several benefits, including greater understanding of disease etiology, improved genetic counseling for family planning, assessment of recurrence risk after kidney transplantation, and opportunities for presymptomatic testing among at-risk family members (5). Conversely, a genetic diagnosis may have emotional consequences, including concern about future health risks and the possibility of transmitting genetic risk to offspring (5, 6). Even among healthy individuals, such as living kidney donor candidates, *APOL1* testing may evoke psychological distress and fear of kidney failure (7). Disease-specific education can mitigate anxiety and improve engagement, as knowledge may foster adaptive illness perceptions and reduce misinformation (8). In addition, *APOL1* testing and disclosure of genotype results have been associated with positive self-reported behavior changes and improved medication adherence among individuals with *APOL1* high-risk genotypes (9).

Despite growing recognition of the genetic and psychosocial dimensions of *APOL1*-associated kidney disease, data on how Black patients perceive and respond to *APOL1* genetic testing remain limited. Pilot studies suggest overall openness to genetic testing (10), yet the nature and evolution of patient-reported concern and worry related to *APOL1* testing have not been well characterized. Understanding these responses is important for addressing potential psychological barriers and supporting informed engagement with precision nephrology. Therefore, we investigated patterns of concern about kidney conditions and worry related to *APOL1* high-risk genotypes among Black adults undergoing *APOL1* genotyping in a prospective cohort study, with the goal of better understanding how genetic risk information may influence perceptions relevant to kidney health and engagement in care.

## Materials and methods

### Study design and participants

We conducted a single-center, prospective study of self-identified Black individuals receiving care through Nephrology and Internal Medicine services at a Midwestern academic medical center (11, 12). Eligibility criteria included self-identified Black race, age ≥18 years, and no dialysis dependence or prior kidney transplantation at enrollment. Potential participants received an explanation of *APOL1* and the current understanding of the relationship between *APOL1* RRVs and CKD. Written informed consent was obtained before genetic testing, and participants received an honorarium for their time at enrollment and survey completion.

Baseline enrollment included blood sample collection for *APOL1* genetic testing as a research test, chart review of demographic and kidney health-related parameters [age, sex, body mass index (BMI), blood pressure, diabetes], and administration of surveys including sociodemographic information and attitudes. Sociodemographic characteristics included age, sex, educational attainment, and annual household income. Estimated glomerular filtration rate (eGFR) was calculated using the 2021 race-free CKD-EPI creatinine equation (13).

Detailed information concerning the study flow and genetic testing is provided in the **Supplementary Material** and **Supplementary Figure 1** (11, 12). Relevant to this report, individual *APOL1* genotyping results were returned to participants in person, together with a written report, by the 3-month follow-up visit. Research results were private information of the participants and were not shared directly with referring clinicians, although participants could share their results with clinicians if they chose to do so.

In addition to education provided by the study team, following the sharing of *APOL1* genotype results, participants were offered genetic counseling with the same certified genetic counselor affiliated with the institution. Counseling was conducted as a separate counseling visit and included education regarding *APOL1*-associated kidney disease risk, interpretation of test results, and discussion of participant questions. Counseling emphasized that an *APOL1* high-risk genotype is a risk factor rather than a diagnosis and that kidney disease is not inevitable. Discussions also highlighted the importance of kidney monitoring and management of other risk factors. Counseling costs were covered by the protocol, and all participants had access to genetic counseling regardless of genotype result.

Participant surveys and chart review of health history and laboratory data were performed at baseline and approximately 3 and 12 months after enrollment, in coordination with standard-of-care follow-up. Enrolled participants who did not return for follow-up at the medical center had the opportunity to complete surveys by email or telephone. The study was approved by the Saint Louis University Institutional Review Board (Protocol ID 29188) and is registered at ClinicalTrials.gov (NCT05656261). Enrollment began on January 24, 2019, and this analysis includes participants who completed 3-month follow-up as of January 1, 2025.

### Concerns and worrying attitudes

The survey was developed by the study team to assess *APOL1*-related perceptions, attitudes, and anticipated behavioral responses and was informed by established behavioral frameworks, including the Health Belief Model, and prior *APOL1*-related literature (14–16). Participants were asked about their degree of concerns regarding kidney conditions and worry about having an *APOL1* high-risk genotype using a survey completed at baseline and 3 months after enrollment. Degree of concern was assessed on a Likert scale ranging from 1 (“not at all”) to 5 (“extremely”). For selected analyses, responses were dichotomized as “very much or extremely concerned” (Likert score 4 or 5) versus all other responses. Worry was assessed as a binary indicator. We examined changes in kidney-related concerns and worry from baseline to 3 months.

### Statistical analysis

Demographic and clinical parameters were summarized according to *APOL1* genotype and presented as percentages for categorical variables and median ± interquartile range (IQR) for continuous variables. Categorical variables were compared using the chi-square test, and continuous variables were compared using the Wilcoxon rank-sum test. *APOL1* RRV distributions were examined according to baseline clinical characteristics. For follow-up analyses, changes in concern and worry from baseline to 3 months were categorized as “decreased,” “unchanged,” or “increased.” *APOL1* RRV distributions were then examined across these response patterns. Differences were considered statistically significant at a two-tailed p-value <0.05. However, statistical significance was not generally anticipated given the sample size.

## Results

**Figure 1:**
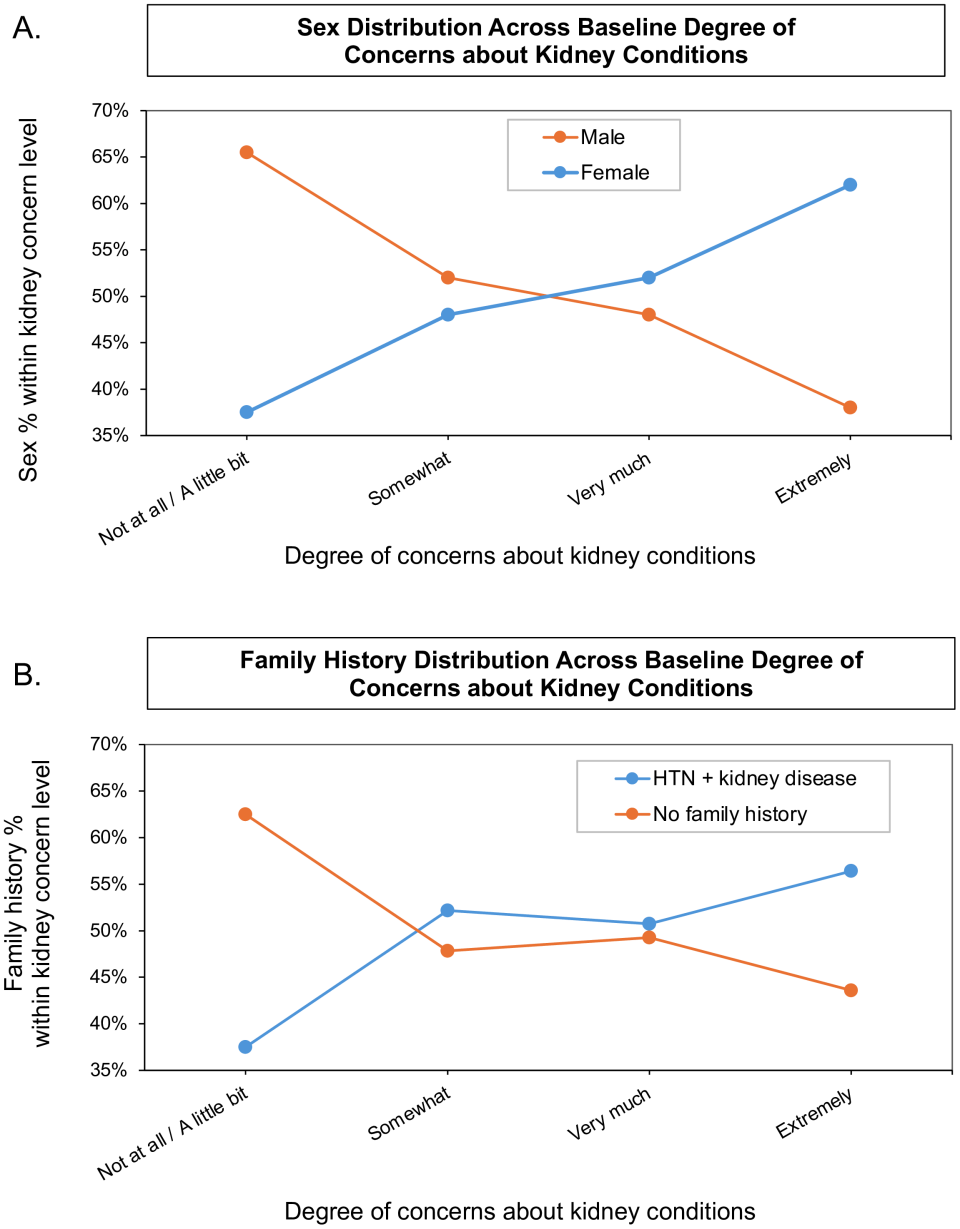
Distributions of participant sex **(A)** and family history **(B)** by baseline degree of concern about kidney conditions.

Among the 200 participants in this study sample, 17.5% (35/200) had an *APOL1* high-risk genotype (2 RRVs) and 82.5% (165/200) had an *APOL1* low-risk genotype (0 or 1 RRV). The median age was 59 years, and 53% (106/200) were women. At baseline, 39% reported being extremely concerned about kidney conditions, 34% very concerned, 12% somewhat concerned, 10% a little concerned, and 6% not concerned at all. Across levels of kidney concern, no differences reached statistical significance, although several trends were observed (**Table 1**). Women comprised 38% of those reporting little or no concern, compared with 52% of those reporting “very much” concern and 62% of those reporting extreme concern, whereas men showed the inverse pattern (**Figure 1A**). A modest gradient was also observed according to *APOL1* genotype, with individuals carrying 2 RRVs representing 6% of the lowest concern group versus approximately 18% to 22% of the moderate- to high-concern groups. Participants reporting extreme concern were slightly younger (median 57 years) than those with moderate concern (62 to 63 years), while body mass index (median 33 kg/m²), blood pressure, and diabetes prevalence (average 20%) were similar across concern categories. A combined family history of hypertension and kidney disease was more common among those with extreme concern (56%) compared with those with low concern (38%) (**Figure 1B**).

**Table 1:** Sociodemographic characteristics and clinical data of the study cohort grouped by baseline concern about kidney conditions.

Baseline factor	Overall (n=200)	Not at all; A little bit (n=32)	Somewhat (n=23)	Very much (n=67)	Extremely (n=78)
*APOL1* genotype, n (%)					
0 RRV	78 (39)	13 (41)	11 (48)	25 (37)	29 (37)
1 RRV	87 (44)	17 (43)	7 (30)	28 (42)	35 (45)
2 RRV	35 (17)	2 (6)	5 (22)	14 (21)	14 (18)
Demographic traits, median (IQR)					
Age, years	59 (17)	60 (17)	63 (23)	62 (19)	57 (15)
Body mass index, kg/m^2^	33 (10)	32 (9)	33 (12)	34 (9)	33 (11)
Sex, n (%)					
Female	106 (53)	12 (38)	11 (48)	35 (52)	48 (62)
Male	94 (47)	20 (62)	12 (52)	32 (48)	30 (38)
Educational attainment, n (%)					
Up to junior high or eighth grade	5 (2)	1 (3)	0 (0)	4 (6)	0 (0)
Some high school, not completed	23 (11)	3 (9)	2 (9)	11 (17)	7 (9)
High school graduate or GED	57 (29)	8 (25)	4 (17)	21 (31)	24 (31)
Some college or technical school	59 (30)	12 (38)	8 (35)	20 (30)	19 (24)
College graduate or greater	53 (27)	7 (22)	9 (39)	10 (15)	27 (35)
Not reported	3 (1)	1 (3)	0 (0)	1 (1)	1 (1)
Annual family income, n (%)					
<$5,000	39 (20)	5 (16)	2 (9)	18 (27)	14 (18)
$5,000 to $15,000	26 (13)	7 (22)	0 (0)	9 (14)	10 (13)
$15,000 to $30,000	30 (15)	5 (16)	5 (22)	10 (15)	10 (13)
$30,000 to $45,000	27 (14)	4 (12)	7 (31)	6 (9)	10 (13)
$45,000 to $60,000	23 (11)	4 (12)	4 (17)	7 (10)	8 (10)
>$60,000	28 (14)	2 (6)	4 (17)	6 (9)	16 (20)
Not reported	27 (13)	5 (16)	1 (4)	11 (16)	10 (13)
Family history, n (%)					
Hypertension alone	72 (36)	12 (38)	8 (35)	25 (37)	27 (35)
Kidney disease alone	6 (3)	2 (6)	1 (4)	1 (1)	2 (3)
Hypertension and kidney disease	102 (51)	12 (38)	12 (52)	34 (51)	44 (56)
Neither	20 (10)	6 (19)	2 (9)	7 (11)	5 (6)
Blood pressure, mmHg, median (IQR)					
Systolic	139 (24)	139 (27)	133 (25)	140 (21)	139 (29)
Diastolic	82 (16)	81 (20)	81 (17)	84 (14)	84 (20)
Diabetes status, n (%)					
Diabetes	40 (20)	6 (19)	5 (22)	16 (24)	13 (17)

Data are presented as column percentage (%).

IQR; interquartile range, GED; general educational development.

**Figure 2:**
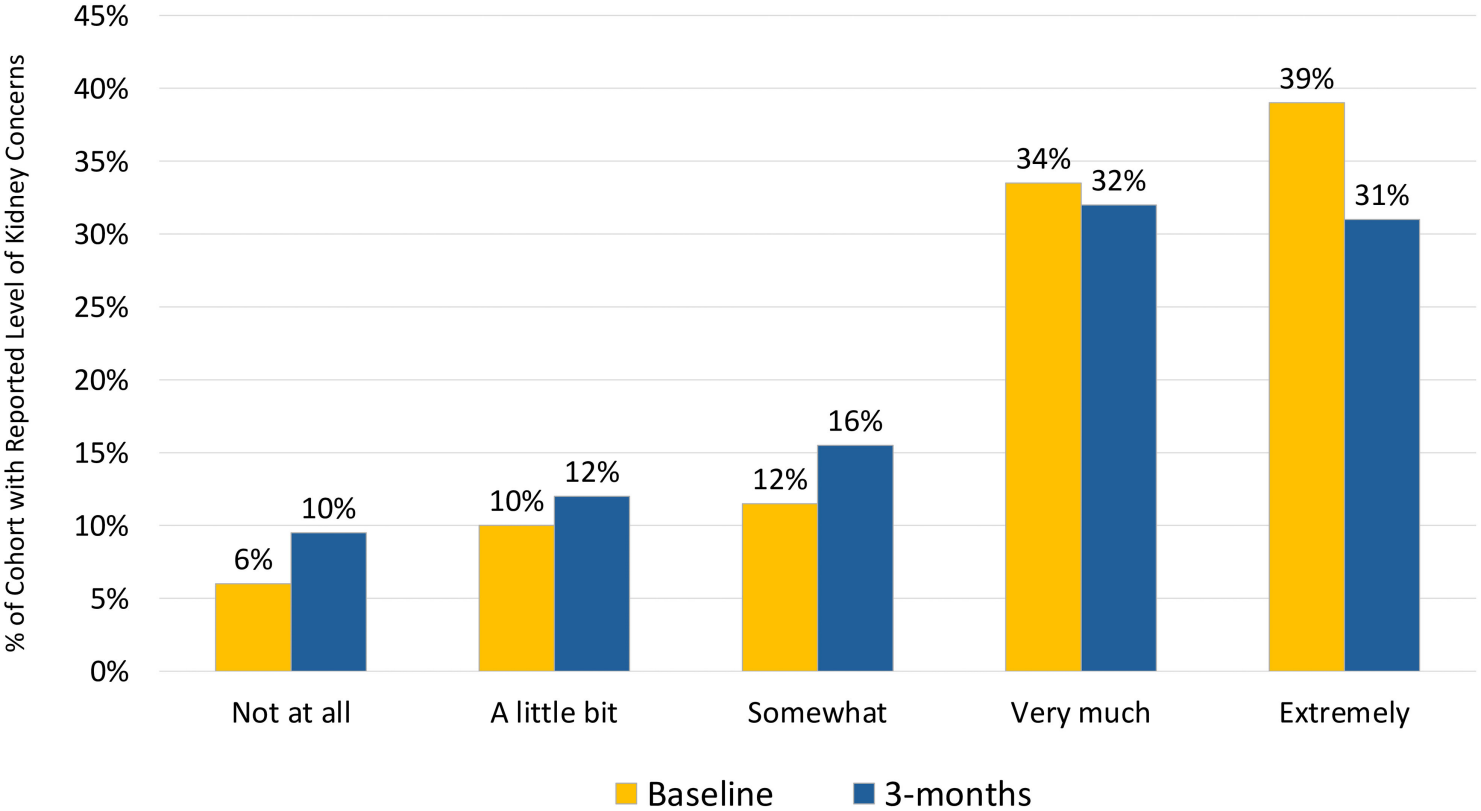
Distributions of the degree of concerns about kidney conditions at baseline and 3 months follow-up in the study cohort.

At 3 months, the proportion of participants who were very much or extremely concerned about kidney conditions declined from 73% (145/200) to 63% (126/200), with a redistribution toward lower and moderate levels of concern (**Figure 2**). The proportion reporting “very much” concern decreased slightly from 34% to 32%, while the proportion reporting extreme concern declined from 39% to 31%. In contrast, lower concern categories (“not at all” or “a little bit”) increased modestly over time.

**Figure 3:**
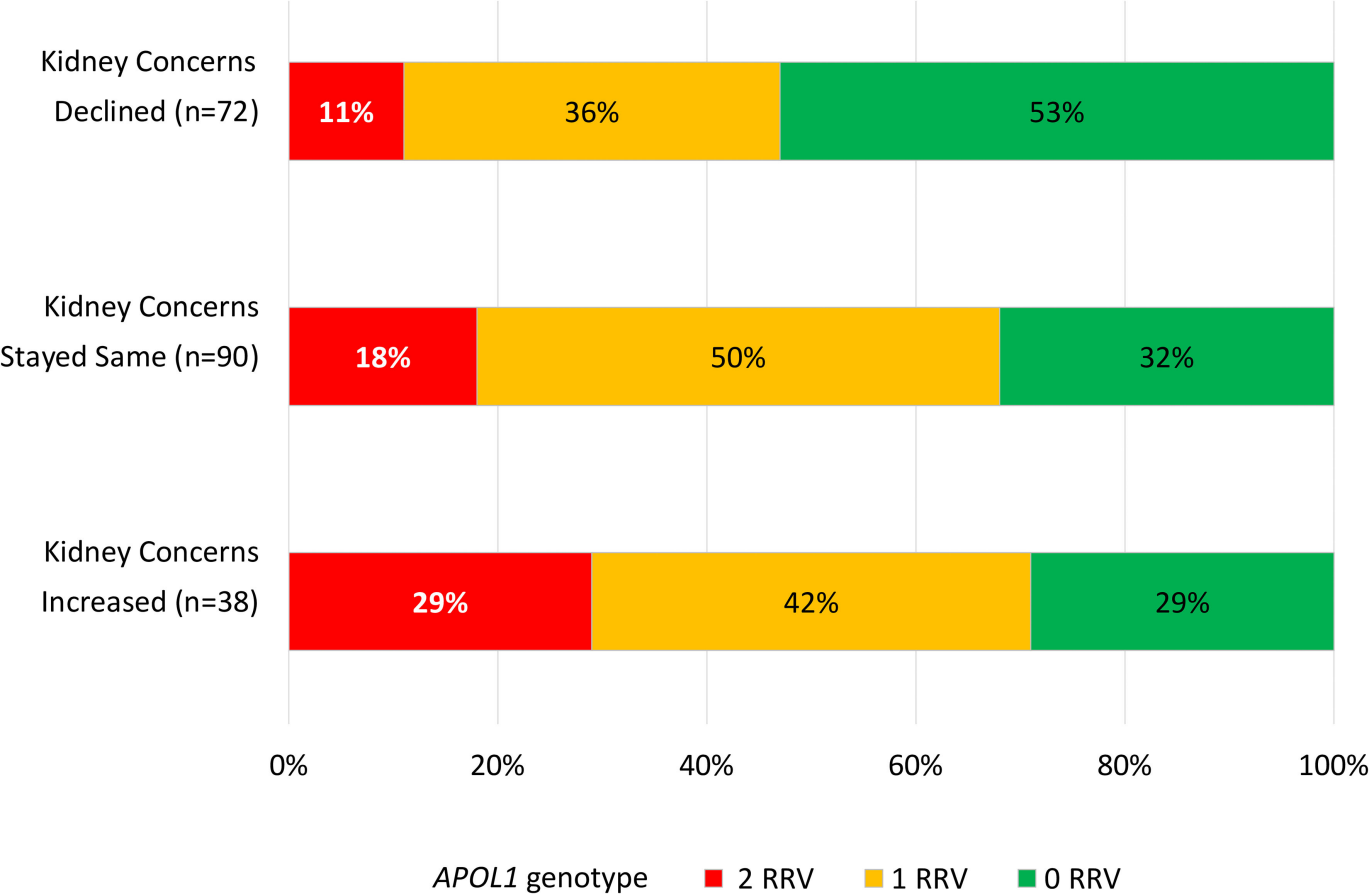
*APOL1* genotype distribution according to the change level in the degree of concerns about kidney conditions from baseline to 3 months. *RRV*, renal risk variant.

At the individual participant level, patient-reported kidney concern decreased in 36% (72/200), remained unchanged in 45% (90/200), and increased in 19% (38/200) at 3 months. An *APOL1* high-risk genotype was more common among participants whose concern increased compared with those whose concern decreased (29% vs 11%; P = 0.02) (**Figure 3**). Changes in kidney concern were not strongly associated with age or sex, which were similarly distributed across groups (**Table 2**). In contrast, higher albuminuria was more common among participants whose concern increased, with 36% having UACR >300 mg/g compared with 14% among those whose concern decreased. Participants with declining concern were more likely to have low albuminuria (<30 mg/g; 58%) (**Table 2**).

**Table 2:** The characteristics of the patients distributed by the change in the degree of concern about kidney condition from baseline to 3 months.

Baseline factor	Declined (n=72)	Stayed same (n=90)	Increased (n=38)
*APOL1* genotype, n (%)			
0 RRV	38 (53)	29 (32)	11 (29)
1 RRV	26 (36)	45 (50)	16 (42)
2 RRV	8 (11)	16 (18)	11 (29)
Age level in years, n (%)			
≤50	19 (26)	22 (25)	11 (29)
51 to 65	34 (48)	40 (44)	16 (42)
>65	19 (26)	28 (31)	11 (29)
Sex, n (%)			
Female	40 (56)	46 (51)	20 (53)
Male	32 (44)	44 (49)	18 (47)
eGFR level in ml/min/1.73 m², n (%)			
≥60	28 (39)	31 (34)	17 (45)
45 to 59	14 (19)	16 (18)	5 (13)
30 to 44	22 (31)	19 (21)	11 (29)
<30	8 (11)	24 (27)	5 (13)
UACR level in mg/g, n (%) *			
<30	25 (58)	22 (43)	11 (44)
30 to 300	12 (28)	14 (28)	5 (20)
>300	6 (14)	15 (29)	9 (36)

*UACR was available for 119 participants.

SD, standard deviation; eGFR, estimated glomerular filtration rate; UACR, Urine albumin/creatinine ratio.

Among the overall cohort, the proportion reporting that an *APOL1* high-risk genotype would be worrying decreased from 14% (28/200) at baseline to 8% (16/200) at 3 months. Among the 16 participants who reported that a high-risk genotype would be worrying at 3 months, 75% (12/16) were women, 75% (12/16) had completed high school or higher education, and 38% (6/16) were in the low-income group. These participants had mild-to-moderately reduced kidney function (median eGFR 48 mL/min/1.73 m²) and macro albuminuria (median UACR 633 mg/g). Only 2 (13%) of participants who reported that a high-risk genotype would be worrying at 3 months had an *APOL1* high-risk genotype, whereas 63% (10/16) had 1 RRV and 25% (4/16) had 0 RRVs.

## Discussion

In this prospective cohort of Black adults undergoing *APOL1* genetic testing at an academic medical center, we evaluated changes in patient-reported kidney-related concerns and worry about genetic risk over 3 months. At baseline, nearly three-quarters of participants reported being very much or extremely concerned about having a kidney problem, underscoring the high prevalence of kidney-related concern among individuals engaged in nephrology and internal medicine care. Although the proportion reporting very much or extreme concerns about kidney conditions declined modestly to 63% at 3 months, concerns remained dynamic at the individual level, with more than one-third of participants experiencing a decrease, nearly half remaining unchanged, and approximately one-fifth reporting increased concerns. Participants whose concerns increased were more likely to carry an *APOL1* high-risk genotype and to have higher levels of albuminuria, suggesting that both genetic and clinical risk factors may influence perceived vulnerability. In parallel, the proportion of participants who reported that an *APOL1* high-risk genotype would be worrying declined from 14% at baseline to 8% at 3 months.

Women represented a larger proportion of participants reporting higher levels of kidney concern and were overrepresented among those who continued to report worry about an *APOL1* high-risk genotype at 3 months. Prior studies across chronic disease populations, including CKD, have shown that women more frequently report health-related concerns, perceived vulnerability, and illness-related distress, which may reflect differences in health awareness, caregiving responsibilities, or willingness to acknowledge and report emotional symptoms (17, 18). Participants with a combined family history of hypertension and kidney disease were also more likely to report extreme levels of kidney concern, consistent with prior work demonstrating that family experience with serious illness heightens perceived susceptibility and threat appraisal (19). Together, these findings underscore the importance of incorporating social context and family experience into patient-centered counseling strategies related to kidney disease risk and *APOL1* genetic testing.

To date, there are limited data regarding concerns about kidney conditions and perceptions of *APOL1* genetic testing among Black Americans. Prior studies have shown that genetic testing can reclassify a prior diagnosis in 11% to 54% of patients with CKD and establish an etiology in 12% to 56% of patients with CKD or end-stage kidney disease without a primary renal diagnosis (20). In 2013, a survey of Black and White first-degree relatives of dialysis patients reported substantial interest in obtaining, using, and sharing genetic test results related to kidney disease (21). Our study extends this work by moving beyond hypothetical interest among relatives to examining concern and worry among individuals who underwent *APOL1* genetic testing as part of a study protocol.

Patient education is critical to helping patients understand their disease course and future care options. Importantly, perceptions about illnesses may also impact individual clinical and physical symptomatology (22). In the current study, more than one-third of participants experienced a decrease in concern about kidney conditions, and the proportion reporting that an *APOL1* high-risk genotype would be worrying declined over the 3-month follow-up period. Participation in a research study that includes access to structured information, engagement with trained clinicians and research staff, and access to formal genetic counseling may help contextualize genetic risk. Future studies should seek to identify which components of education and counseling are most effective in addressing kidney-related concerns and responses to genetic risk information.

*APOL1* RRVs originated in populations with African ancestry and, through migration, are now found among individuals throughout North, Central, and South America (23). Given the impacts of structural racism, medical mistrust, and underrepresentation of Black individuals in clinical trials, it is essential to incorporate patient perspectives and attitudes into *APOL1* testing practices (11). Prior work has shown that concern about psychological distress and receiving “bad news” can serve as a barrier to genetic testing (24), while other studies have demonstrated that disclosure of *APOL1* results may motivate positive lifestyle changes and improved medication adherence among individuals with high-risk genotypes (9). Our findings contribute to this growing body of literature by demonstrating that kidney-related concern is common and dynamic and may vary according to genetic and clinical risk factors.

This study has limitations that warrant consideration. The sample size was modest, and follow-up duration was relatively short, limiting the ability to draw conclusions regarding long-term psychological responses to *APOL1* testing. We did not assess baseline psychiatric conditions, which may have influenced reported concern or worry and limited interpretation of individual responses. Participants were engaged in established medical care, primarily nephrology, and may not represent individuals without regular healthcare access. Information regarding the number of individuals who declined *APOL1* testing and reasons for declining testing was not systematically collected as part of the study. Because the survey instrument was not formally validated, findings regarding concerns and worry should be interpreted as exploratory assessments of participant perceptions rather than validated measures of psychological outcomes. Additionally, changes in concerns and worry over time may reflect both study engagement and broader external influences, including increased awareness of *APOL1*-associated kidney disease and the evolving role of *APOL1* testing within precision nephrology (1). The clinical significance of patient-reported concerns and worry remains uncertain. These responses may reflect adverse psychological effects, increased health awareness and engagement, or a combination of both. Ongoing work is needed in larger and more diverse populations, with longer follow-up and validated psychological, behavioral, and clinical outcome measures, to better understand how *APOL1* testing affects patients over time and how counseling and education can best support informed care.

In summary, in this prospective cohort of Black adults undergoing *APOL1* genotyping, patient-reported kidney-related concerns were common and generally persisted over time, while patient-reported worry related to *APOL1* genetic risk declined. Participants whose kidney concerns increased over time were more likely to have an *APOL1* high-risk genotype and higher levels of albuminuria. These findings highlight the nuanced psychological responses associated with *APOL1* testing and underscore the importance of patient-centered education and counseling as *APOL1* testing becomes increasingly integrated into clinical practice.

## Data Availability

Data are available in summary form due to limitations of the IRB. Further inquiries can be directed to the corresponding author/s.

## St. Louis *APOL1* study collaborators

Orhun Aydin, PhD; Anne Buganski, RN; Ezequiel Bellorin Font, MD; Ava DeLonais-Parker, BS; Farzana Hoque, MD; Nourhan Houjeij, MD; Kevin Lee, MPA; Mary Lesko, RN; Kenan Li, PhD; Kunal Malhotra, MD; Rushda Mansuri, MD, MPH; Marilyn Maxwell, MD; Enbal Shacham, PhD; and Mowaffaq Said, MD.

## Supplementary material

Supplementary Figure 1 Schematic presentation of study procedures. *APOL1*, apolipoprotein L1; CKD, chronic kidney disease; CLIA, Clinical Laboratory Improvement Amendments; DNA, deoxyribonucleic acid; RRV, renal risk variant; SNP, single nucleotide polymorphism. Created in BioRender. Lentine, K. (2026) https://BioRender.com/qtws5mi.
