# Supplementary material for: Beyond the test: patient-reported concerns and worries regarding kidney conditions and APOL1 genetic testing among Black patients
Source: Front Nephrol. 2026 Jul 22;6:1815147. doi: 10.3389/fneph.2026.1815147 (PMC13438459; doi:10.3389/fneph.2026.1815147)
Supplement: Supplementary file 1 [file DataSheet1.docx]

Genetic Testing

After the baseline survey at enrollment, blood samples were drawn and sent to the CLIA-approved lab at Mid America Transplant for *APOL1* genotyping. Two single nucleotide polymorphisms (SNPs) in the *APOL1* G1 renal-risk allele (rs73885319; rs60910145) and an insertion/deletion for the G2-renal-risk allele (rs143830837) were genotyped using a custom assay designed at Wake Forest University School of Medicine on the Sequenom platform (San Diego, California).^1^ *APOL1* genotype results were expressed as 0 *APOL1* RRV (wildtype), 1 *APOL1* RRV (G1 or G2 allele), or 2 *APOL1* RRVs.^2^ An *APOL1* low-risk genotype was defined as the presence of 0 or 1 RRV, meaning no variant (G0/G0) or a monoallelic variant (G0/G1 or G0/G2). *APOL1* high-risk genotypes were defined as the presence of 2 RRVs, meaning biallelic variants (G1/G1, G2/G2, or G1/G2). Patients with *APOL1* high-risk genotypes were counseled by a nephrologist on the study team and were also offered additional genetic counseling sessions with a genetic counselor at our center. Research results were returned to the participants before the second survey. Results were confidential and were not entered into the medical record, but patients could discuss with their treating physician if they chose to do so.

**REFERENCES**

1.	Freedman BI, Julian BA, Pastan SO*, et al.* Apolipoprotein L1 gene variants in deceased organ donors are associated with renal allograft failure. *Am J Transplant* 2015; **15:** 1615-1622.

2.	Dahl NK, Bloom MS, Chebib FT*, et al.* The Clinical Utility of Genetic Testing in the Diagnosis and Management of Adults with Chronic Kidney Disease. *J Am Soc Nephrol* 2023; **34:** 2039-2050.
